# Age-associated DNA methylation changes in immune genes, histone modifiers and chromatin remodeling factors within 5 years after birth in human blood leukocytes

**DOI:** 10.1186/s13148-015-0064-6

**Published:** 2015-03-26

**Authors:** Nathalie Acevedo, Lovisa E Reinius, Morana Vitezic, Vittorio Fortino, Cilla Söderhäll, Hanna Honkanen, Riitta Veijola, Olli Simell, Jorma Toppari, Jorma Ilonen, Mikael Knip, Annika Scheynius, Heikki Hyöty, Dario Greco, Juha Kere

**Affiliations:** Department of Medicine Solna, Translational Immunology Unit, Karolinska University Hospital, Stockholm, Sweden; Department of Biosciences and Nutrition, Center for Innovative Medicine, Karolinska Institutet, Stockholm, Sweden; Department of Biology, Bioinformatics Centre, University of Copenhagen, Copenhagen, Denmark; Unit of Systems Toxicology, Finnish Institute of Occupational Health, Helsinki, Finland; Department of Virology, School of Medicine, University of Tampere, Tampere, Finland; Department of Pediatrics, Oulu University Hospital, University of Oulu, Oulu, Finland; Department of Pediatrics, Turku University Hospital, Research Centre of Applied and Preventive Cardiovascular Medicine, University of Turku, Turku, Finland; Department of Physiology and Pediatrics, Turku University Hospital, University of Turku, Turku, Finland; Immunogenetics Laboratory, University of Turku, Finland and Department of Clinical Microbiology, University of Eastern Finland, Kuopio, Finland; Children’s Hospital, Helsinki University Central Hospital, University of Helsinki, Helsinki, Finland; Department of Paediatrics, Tampere University Hospital, Tampere, Finland; Fimlab Laboratories, Tampere, Finland; Folkhälsan Institute of Genetics, Helsinki, and Research Programs Unit, University of Helsinki, Helsinki, Finland

**Keywords:** Age-modified CpG, Childhood, DNA methylation, Genes, Leukocytes, Longitudinal

## Abstract

**Background:**

Age-related changes in DNA methylation occurring in blood leukocytes during early childhood may reflect epigenetic maturation. We hypothesized that some of these changes involve gene networks of critical relevance in leukocyte biology and conducted a prospective study to elucidate the dynamics of DNA methylation. Serial blood samples were collected at 3, 6, 12, 24, 36, 48 and 60 months after birth in ten healthy girls born in Finland and participating in the Type 1 Diabetes Prediction and Prevention Study. DNA methylation was measured using the HumanMethylation450 BeadChip.

**Results:**

After filtering for the presence of polymorphisms and cell-lineage-specific signatures, 794 CpG sites showed significant DNA methylation differences as a function of age in all children (41.6% age-methylated and 58.4% age-demethylated, Bonferroni-corrected *P* value <0.01). Age-methylated CpGs were more frequently located in gene bodies and within +5 to +50 kilobases (kb) of transcription start sites (TSS) and enriched in developmental, neuronal and plasma membrane genes. Age-demethylated CpGs were associated to promoters and DNAse-I hypersensitivity sites, located within −5 to +5 kb of the nearest TSS and enriched in genes related to immunity, antigen presentation, the polycomb-group protein complex and cytoplasm.

**Conclusions:**

This study reveals that susceptibility loci for complex inflammatory diseases (for example, *IRF5*, *NOD2*, and *PTGER4*) and genes encoding histone modifiers and chromatin remodeling factors (for example, *HDAC4*, *KDM2A*, *KDM2B*, *JARID2*, *ARID3A*, and *SMARCD3*) undergo DNA methylation changes in leukocytes during early childhood. These results open new perspectives to understand leukocyte maturation and provide a catalogue of CpG sites that may need to be corrected for age effects when performing DNA methylation studies in children.

**Electronic supplementary material:**

The online version of this article (doi:10.1186/s13148-015-0064-6) contains supplementary material, which is available to authorized users.

## Background

Methylation of cytosines to 5-methylcytosines in the context of CpG dinucleotides is an important epigenetic modification that regulates gene expression and cell-specific functions. Some DNA methylation signatures are maintained during mitosis and contribute to the so-called ‘epigenetic memory’, which determine cell lineage. Other DNA methylation patterns are very dynamic, change during lifetime and mediate several physiological events such as cell differentiation, cell maturation and tissue-specific gene expression [1,2]. From early developmental stages through senescence, CpG sites are methylated by DNA methyltransferases (DNMT3a/DNMT3b and DNMT1) [3] and demethylated either passively or by active mechanisms implicating 5-hydroxymethylation, ten-eleven translocator (TET) proteins and thymidine glycosidases [4,5]. Studies in diverse human tissues have demonstrated that DNA methylation levels are modified as a function of age [6-10]. Indeed, it is possible to predict the age of a tissue based on its methylation signatures on a broad number of CpG sites [6,9,11-13]. Most studies investigating age-associated DNA methylation changes have been performed in adults and from the perspective of cell senescence, longevity, cancer, stem cell functions and chronological age [12,14-19]. Still, few studies have documented the dynamics of DNA methylation during early childhood [20-23].

It is known that increasing age leads to genome-wide demethylation in transposable repetitive elements (including Alu and L1) as well as in gene coding regions [19,24,25]. Increasing age is also associated to increased methylation of certain CpGs in specific gene families, CpG islands [26], polycomb (PcG) target genes [27] and promoters with bivalent chromatin domains [28]. Age-associated changes in DNA methylation have been implicated in tumour development and certain chronic diseases [29]. The recognition of age-modified CpG sites in infants is essential to identify genes that might be epigenetically modified during this period of life and, if disturbed, might contribute to the susceptibility to complex inflammatory diseases in childhood. The identification of age-modified CpG sites during early childhood is also important, because early exposure to environmental factors such as pollutants and pesticides might alter the methylation levels of inflammatory genes and these signatures may be sustained during years, possibly predisposing to disease [30,31]. The aims of this study were the following: 1) to identify CpG sites with longitudinal changes in DNA methylation levels within 3 to 60 months after birth in healthy children and 2) to annotate the genomic distribution and functional relationships of age-modified CpG sites during early childhood. The present study provides a catalogue of 794 age-modified CpG sites that robustly reflect the changes in DNA methylation levels that occur in human blood leukocytes within 3 to 60 months after birth. Notably, we found that the genomic location of age-modified CpG sites differs depending whether the CpGs become age methylated or age demethylated. The functional annotation of the genes containing age-modified loci indicated that methylation changes related to age may not be due only to a stochastic DNA methylation drift but rather correspond to a programme with potential functional relevance in leukocyte biology during this period of life.

## Results

**Figure 1:**
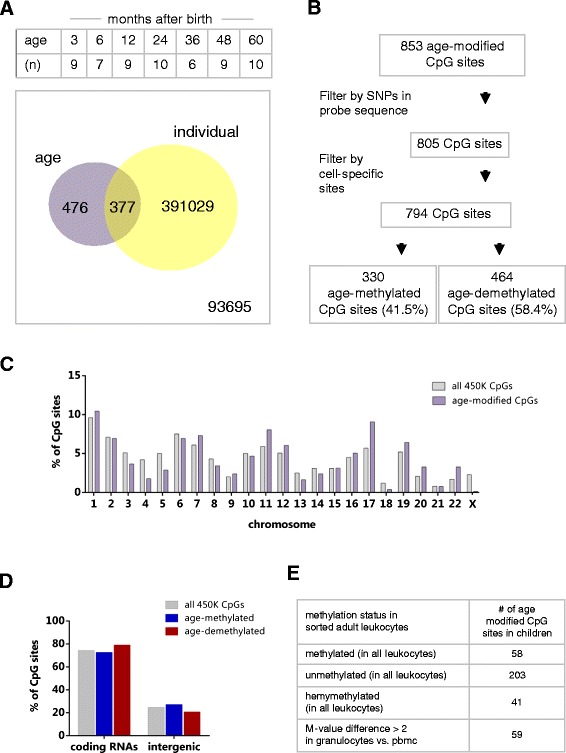
**Descriptive information of age-modified CpG sites. (A)** Schema showing the time points analysed, number of samples (*n*) and the number of differentially methylated CpGs based on age and individual. **(B)** Filtering steps on the 853 age-modified CpGs. **(C)** Chromosomal distribution of age-modified CpGs in relation to the expected proportions according to the location of all probes in the 450 K assay. **(D)** Distribution of age-modified CpG sites within RNA coding regions or intergenic regions in relation to the expected proportions of all probes in the 450 K assay. **(E)** Number of age-modified CpG sites that were found homogeneously methylated in seven populations of sorted blood leukocytes, granulocytes and peripheral blood mononuclear cells (PBMCs) from healthy adults as described in [34]. The list of age-modified CpG sites with homogeneous methylation in sorted leukocytes is presented in Additional file 1.

We analysed the longitudinal changes in DNA methylation in a total of 60 samples at 3, 6, 12, 24, 36, 48 and 60 months after birth, using serial DNA samples extracted from peripheral blood leukocytes of ten healthy girls participating in the Finnish Type 1 Diabetes Prediction and Prevention Study (DIPP) (Table 1). DNA methylation levels were measured in 485.577 CpG sites distributed in 99% of the annotated RefSeq genes using the HumanMethylation450 BeadChip (Illumina, San Diego, CA, USA) [32]. DNA methylation levels were log_2_ transformed to M values and then statistically evaluated using *limma* [33]. A single procedure consisting of two steps was used to infer the association between age and DNA methylation. In the first step, a linear model was used considering the age and the individual (repeated samples from the same person); the study of the variance was performed but no list of differentially methylated probes was generated. Then, the information on the variance was utilized as prior for the second step of the analysis, which consisted of a moderated *t*-test carried out comparing the DNA methylation in samples at 3 months vs the samples at 60 months. We found 853 CpG sites with significant differential methylation due to age (Bonferroni-corrected *P* value <0.01). Of these, 476 CpGs were exclusively affected by age and 377 CpGs were affected by both age and individual (Figure 1A). Since single nucleotide polymorphisms (SNPs) in the probe sequence may affect methylation measurements, all age-modified CpG sites containing a SNP within the probe with a minor allele frequency (MAF) above 0.01 in the Finnish population were filtered out (*n* = 48). Moreover, to avoid the confounding effects of CpG sites that are differentially methylated among leukocyte populations due to cell lineage (cell specific), the 853 age-modified CpG sites were contrasted against a list of 2,228 CpG sites with significant differential DNA methylation in sorted leukocytes [34], which serve as cell-type classifiers. Eleven age-modified CpG sites were found in this list and therefore excluded. After these filtering steps, 794 age-modified CpG sites remained for further analyses (330 age-methylated sites and 464 age-demethylated sites) (Figure 1B). The detailed list of age-modified CpG sites and fold changes of M values and *P* values is found in Additional file 1.

**Table 1:** **Descriptive information on the study individuals (** ***n*** **= 10)**

**Child number**	**Date of birth**	**HLA-DR-DQ haplotype**	**Risk class** ^**a**^	**Mode of delivery**	**Maternal smoking during pregnancy**	**Age at end of exclusive breast-feeding (months)**	**Age at end of total breast-feeding (months)**	**Samples (time points) included in the analysis after QC**
1	2000.03.21	DRB1*04:01-DQB1*03:02/DRB1*04:04-DQB1*03:02	3	Caesarean section	No	-	<3	3 m, 12 m, 24 m, 36 m, 48 m, 60 m
2	2000.04.10	DRB1*04:04-DQB1*03:02/(DR1/10)-DQB1*05:01	3	Caesarean section	No	2.2	3.5	24 m, 60 m
3	2002.04.18	DRB1*04:04-DQB1*03:02/(DR1/10)-DQB1*05:01	3	Vaginal	No	<5	7 to 11	3 m, 6 m, 12 m, 24 m, 48 m, 60 m
4	2002.05.16	DRB1*04:04-DQB1*03:02/(DR7)-DQA1*02:01-DQB1*03:03	1	Vaginal	Yes	0.2	10.5	3 m, 6 m, 12 m, 24 m, 48 m, 60 m
5	2002.08.04	DRB1*04:01-DQB1*03:02/DRB1*04:04-DQB1*03:02	3	Vaginal	No	5.0	10.0	3 m, 6 m, 12 m, 24 m, 36 m, 48 m, 60 m
6	2002.08.21	DRB1*04:04-DQB1*03:02/(DR9)-DQA1:03-DQB1*03:03	3	Vaginal	No	<3	13 to 17	3 m, 12 m, 24 m, 48 m, 60 m
7	2002.10.04	DRB1*04:01-DQB1*03:02/ (DR8)-DQB1*04	3	Vaginal	No	3.0	8.0	3 m, 6 m, 12 m, 24 m, 36 m, 48 m, 60 m
8	2002.10.29	DRB1*04:01-DQB1*03:02/(DR1/10)-DQB1*05:01	3	Caesarean section	No	<3	8 to 11	3 m, 6 m, 12 m, 24 m, 36 m, 48 m, 60 m
9	2002.11.20	DRB1*04:03-DQB1*03:02/(DR13)-DQB1*06:03	0	Vaginal	No	5.5	9.0	3 m, 6 m, 12 m, 24 m, 36 m, 48 m, 60 m
10	2002.11.21	DRB1*04:04-DQB1*03:02/(DR1/10)-DQB1*05:01	3	Vaginal	No	2.0	2.7	3 m, 6 m, 12 m, 24 m, 36 m, 48 m, 60 m

^a^Risk for T1D classified in five classes from decreased risk (0) to strongly increased risk (4) as presented in Hekkala *et al* [50].

HLA = human leukocyte antigen; m = months.

Age-modified CpG sites were found in all autosomes with frequencies that correlated with the distribution of probes in the assay (*r* = 0.86, *P* < 0.0001, Figure 1C) except for the X chromosome which had only one age-modified CpG site in the 5′UTR of the gene encoding claudin 2 (chrX: 106161451, p_bonf_ = 3.34 × 10^−9^). Considering that this chromosome contains 11,232 of all tested probes (2.3%), our finding reproduces previous observations suggesting that the X chromosome is ‘reluctant’ to methylation changes over time [20,22]. Furthermore, age-modified CpG sites were most frequently located in RNA coding genes than in intergenic regions. There were no deviations from the expected proportions according to the distribution of probes in the 450 K assay between age-methylated and age-demethylated sites (Figure 1D).

The effects of age on the DNA methylation levels of these sites were supported by the identification of genes having at least two age-modified CpG sites (range two to six sites) spanning over stretches of sequence from few base pairs (bp) up to kilobases (mean 19.7 ± 51.1 kb). If at least two CpG sites showed the same methylation trend in a given loci, they configure an age-modified region. Nowadays, the length of a differentially methylated region or the number of CpG sites that they should contain is debated; therefore in the present study, we adopted this more global definition to consider a broader sequence length and the tendency of the age effects. Genes containing age-methylated regions are presented in Table 2, and genes containing age-demethylated regions are presented in Table 3. Further support on these findings was suggested by the detection of age-modified CpG sites in genes belonging to the same families but encoded on separate chromosomes, for instance the homeobox cluster A on chromosome 7p15.2 (*HOXA3* and *HOXA10*) and the homeobox cluster B on chromosome 17q21.3 (*HOXB6*) (Additional files 1 and 2).

**Table 2:** **Age-methylated regions within 3 to 60 months after birth in blood leukocytes**

**Gene symbol**	**Gene name**	**Function**	**Locus**	**Number of CpGs**	**Illumina ID**	**Region length (bp)**
*TTC22*	Tetratricopeptide repeat domain 22	Mediate protein-protein interactions and chaperone activity	1p32.3	3	cg24550149	454
					cg15645660	
					cg11949335	
*SPEG*	SPEG complex locus	Myocyte cytoskeletal development and marker of differentiated vascular smooth cells	2q35	3	cg14074251	51,924
					cg26530345	
					cg02557933	
*SNED1*	Sushi, nidogen and EGF-like domains 1	Membrane-bound signalling molecule; hormonal regulation	2q37.3	5	cg02532017	34,671
					cg07644939	
					cg25241559	
					cg19075225	
					cg17053285	
*TRIM7*	Tripartite motif containing 7	Ubiquitin protein ligase; Initiation of glycogen synthesis	5q35.3	2	cg17279652	170
					cg26600753	
*DDR1*	Discoidin domain receptor tyrosine kinase 1	Regulation of cell growth, differentiation and metabolism; cell communication with environment	6p21.3	3	cg16215084	238
					cg00934322	
					cg09965419	
*TNXB*	Tenascin XB	Anti-adhesive effect; matrix maturation during wound healing	6p21.3	3	cg19071976	51,088
					cg16662408	
					cg02657865	
*MAD1L1*	MAD1 mitotic arrest deficient-like 1	Mitotic spindle-assembly checkpoint; cell cycle control and tumour suppression	7p22	6	cg13700912	182,950
					cg06555468	
					cg19513987	
					cg16026522	
					cg09174162	
					cg00963171	
*UPP1*	Uridine phosphorylase 1	Phosphorolysis of uridine to free bases and ribose-1-phosphate	7p12.3	3	cg14983135	170
					cg10317717	
					cg21484940	
*ZNF503*	Zinc finger protein 503	TF; transcriptional regulation; neural precursor cell proliferation	10q22.2	2	cg13997975	553
					cg03487027	
*DGKZ*	Diacylglycerol kinase, zeta	Kinase; regulate diacylglycerol levels in intracellular signal transduction	11p11.2	2	cg18908017	3,940
					cg09802018	
*B4GALNT1*	Beta-1,4-N-acetyl-galactosaminyl transferase 1	Biosynthesis of G(M2) and G(D2) glycosphingolipids	12q13.3	2	cg09932758	1,936
					cg25663970	
*BTBD11*	BTB (POZ) domain containing 11	Transcription cofactor; Protein heterodimerization activity (?)	12q23.3	3	cg27567561	566
					cg13935577	
					cg01478234	
*TEPP*	Testis, prostate and placenta expressed	Unknown	16q21	2	cg12499872	91
					cg00491255	
*CNTNAP1*	Contactin-associated protein 1	Recruitment and activation of intracellular signalling pathways in neurons	17q21	2	cg16308533	39
					cg11629889	
*TBCD*	Tubulin folding cofactor D	Folding of beta-tubulin	17q25.3	2	cg16555866	35,310
					cg00663986	
*NFIX*	Nuclear factor I/X CCAAT-binding transcription factor	Transcription factor (TF)	19p13.3	4	cg06458248	9,812
					cg27392771	
					cg01634146	
					cg10767662	
*LRFN1*	Leucine-rich repeat and fibronectin type III domain containing 1	Promotes neurite outgrowth in hippocampal neurons. Regulates and maintain excitatory synapses	19q13.2	2	cg26910511	100
					cg25156118	
*TMC2*	Transmembrane channel-like 2	Ion channel; expression in the inner ear suggests that it may be crucial for normal auditory function	20p13	3	cg12233487	146
					cg23648082	
					cg03243506	
*CLDN5*	Claudin 5	Claudin (physical barrier to solutes); membrane protein and tight junctions	22q11.21	2	cg04463638	366
					cg14553765

**Table 3:** **Age-demethylated regions within 3 to 60 months after birth in blood leukocytes**

**Gene symbol**	**Gene name**	**Function**	**Locus**	**Number of CpGs**	**Illumina ID**	**Region length (bp)**
*PRDM16*	PR domain containing 16	TF; zinc finger transcription factor (KRAB box)	1p36.23-p33	4	cg17001566	249,737
					cg12436196	
					cg01418153	
					cg03254465	
*CITED4*	Cbp/p300-interacting transactivator, with Glu/Asp-rich carboxy-terminal domain, 4	Transcriptional co-activator; CBP and p300 binding; co-activator of AP2	1p34.2	2	cg08719289	42
					cg10705800	
*ATOH8*	Atonal homolog 8	TF, DNA binding, transcriptional regulation; nuclease	2p11.2	3	cg05318142	13,349
					cg08079596	
					cg05584950	
*HDAC4*	Histone deacetylase 4	Histone deacetylase; reductase; transcriptional repression when tethered to a promoter	2q37.3	3	cg05870586	362
					cg15058210	
					cg05903736	
*CLEC3B*	C-type lectin domain family 3, member B (tetranectin)	Extracellular matrix structural protein	3p22-p21.3	3	cg02396676	224
					cg22505962	
					cg06117855	
*B3GALT4*	UDP-Gal: betaGlcNAc beta 1,3-galactosyltransferase, polypeptide 4	Glycosyltransferase; synthesis of type 1 carbohydrate chains. Biosynthesis of ganglioseries glycolipid.	6p21.3	2	cg17103217	172
					cg06362282	
*NFE2L3*	Nuclear factor (erytroid-derived 2)-like 3	TF; binding of antioxidant response elements in target genes.	7p15.2	2	cg14684457	143
					cg10536999	
*CUX1*	Cut-like homeobox 1	TF; DNA binding protein. Regulate gene expression, morphogenesis, differentiation and cell cycle progression	7q22.1	3	cg10692693	82
					cg05910443	
					cg03310939	
*NACC2*	NACC family member 2, BEN and BTB (POZ) domain containing	Histone deacetylase	9q34.3	2	cg14147151	37,942
					cg14126392	
*BLOC1S2*	Biogenesis of lysosomal organelles complex-1, subunit 2	Dehydrogenase; formation of lysosome-related organelles	10q24.31	2	cg26610808	5
					cg15298486	
*HCCA2*	YY1-associated protein 1	TF	11q22	3	cg01469847	13,771
					cg20973931	
					cg12007048	
*ADRBK1*	Adrenergic, beta, receptor kinase 1	Phosphorylation of beta-2-adrenergic receptor	11q13.1	2	cg13924996	100
					cg11436362	
*SHANK2*	SH3 and multiple ankyrin repeat domains 2	Molecular scaffold in the postsynaptic density	11q13.2	2	cg11155924	68,036
					cg27643147	
*PSTPIP1*	Proline-serine-threonine phosphatase interacting protein 1	CD2 binding protein. CD2-triggered T cell activation; membrane trafficking regulatory protein	15q24.3	2	cg26227523	1,804
					cg21322248	
*GPRC5C*	G protein-coupled receptor, family C, group 5, member C	G-protein coupled receptor; cellular effects of retinoic acid (?)	17q25	3	cg12776171	157
					cg26663490	
					cg16120833	
*MGAT5B*	Mannosyl (alpha-1,6-)-glycoprotein beta-1,6-N-acetyl glucosaminyltransferase, isoenzyme B	Synthesis of complex cell surface N-glycans	17q25.2	2	cg23838005	66
					cg05514299	
*ARID3A*	AT-rich interactive domain 3A (BRIGHT-like)	TF; cell lineage regulation; cell cycle control; chromatin structure modification	19p13.3	4	cg12713583	6,988
					cg02001279	
					cg18598117	
					cg01774027	
*TEF*	Thyrotrophic embryonic factor	TF	22q13.2	2	cg20534570	419
					cg13228442	
*TSPO*	Translocator protein (18 kDa)	Steroid hormone synthesis	22q13.31	2	cg00343092	722
					cg08909806	

Since age-modified CpG sites were detected in whole blood, we further investigated their cell-type specific annotations according to the Illumina manifest. First, none of the 794 age-modified CpG sites was annotated to known tissue-specific differentially methylated regions (t-DMR). However, 12 age-modified CpG sites were annotated to cancer-specific DMR (c-DMR) and 62 CpG sites to reprogramming-specific DMRs (r-DMR) [35]. Based on the regulatory feature group, 15.8% of the age-modified CpGs were annotated as gene-associated cell-type specific (*n* = 8), promoter-associated cell-type specific (*n* = 17) and unclassified cell-type specific (*n* = 101), Additional file 1. We also evaluated the DNA methylation levels of age-modified CpG sites in a dataset of sorted blood leukocytes from male adults [34]. Interestingly, 38% of 794 age-modified CpG sites identified in this study showed homogeneous DNA methylation in sorted leukocytes, granulocytes and peripheral blood mononuclear cells from healthy adults (Figure 1E and Additional file 1); suggesting that at least these age-modified CpG sites may not be lineage specific and that it is unlikely that the detected age effects would be a result of differences in cell composition. In contrast, 7.4% of all the age-modified CpG sites had a difference of at least two units in M value between the mononuclear fraction and the granulocyte fraction (Figure 1E), suggesting that methylation at those age-modified CpG sites is much variable between mononuclear cells and granulocytes, and therefore they are more susceptible to be affected by cell heterogeneity.

### The genomic distribution of age-modified CpG sites

**Figure 2:**
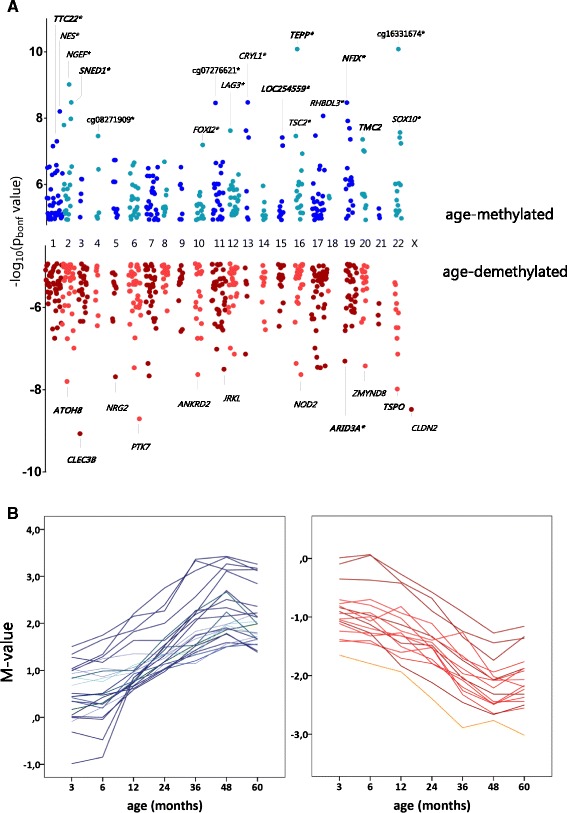
**Chromosomal distribution and DNA methylation trends of the significant age-modified CpG sites. (A)** Dot plot showing the chromosomal distribution of age-methylated CpGs (blue dots) and age-demethylated CpGs (red dots) in relation to the Bonferroni-corrected *P* value. For methylated genes: *TTC22* = tetratricopeptide repeat domain 22; *NES* = nestin; *NGEF* = neuronal guanidine nucleotide exchange factor; *SNED1* = sushi nidogen and EGF-like domains 1; *FOXI2* = forkhead box I2; *LAG3* = lymphocyte activation gene 3; CRYL1 = crystallin lambda 1; *TEPP* = testis prostate and placenta expressed; *TSC2* = tuberous sclerosis 2; *RHBDL3* = rhomboid, veinlet-like 3 (*Drosophila*); *NFIX* = nuclear factor I/X; *TMC2*: transmembrane channel-like 2; *SOX10* = SRY-box 10. For demethylated genes: *ATOH8* = atonal homolog 8; *CLEC3B* = C-type lectin domain family 3, member B, *NRG2* = neuregulin 2; *PTK7* = protein tyrosine kinase 7; *ANKRD2* = ankyrin repeat domain 2; *JRKL* = JRK-like; *NOD2* = nucleotide-binding oligomerization domain containing 2; *ARID3A* = AT-rich interactive domain 3A; *ZMYND8* = zinc finger, MYND-type containing 8; *TSPO* = translocator protein (18 kDa); *CLDN2* = claudin 2. An asterisk next to the gene symbol indicates that the age-modified CpG site has similar DNA methylation levels in sorted blood leukocytes of healthy adults. Genes in bold indicate that the annotated CpG site is embedded in an age-modified region. Detailed information on *P* values is presented in Additional file 1. **(B)** Time trends in DNA methylation (M value) for age-methylated sites (blue) and age-demethylated sites (red). M values above 1 represent that the site is methylated, and M values below −1 represent that the site is demethylated. A value of 0 is proportional to a beta value of 0.50. Each line represents a CpG site.

The chromosomal distribution of the age-modified CpG sites according to their Bonferroni-corrected *P* value (p_bonf_) is presented in Figure 2A. Genes containing the most significant age-modified CpG sites in peripheral blood leukocytes within 5 years after birth are annotated in the figure (p_bonf_ below 6.5 × 10^−8^). The Illumina identifier is presented for three age-methylated CpG sites without any transcripts mapped to their position (intergenic), including the most significant age-modified CpG at chr. 22:28074071 (cg16331674, p_bonf_ = 8.1 × 10^−11^). The majority of the top significant age-methylated CpG sites were also homogeneously methylated in sorted peripheral blood leukocytes from healthy adults (showed with an asterisk in Figure 2A). Furthermore, we found that many of the top significant age-modified CpG sites were embedded into age-modified regions (see Figure 2A, Tables 2 and 3). Examples of the time trends for age effects on DNA methylation in methylated and demethylated sites are presented in Figure 2B. Overall, the kinetics of the DNA methylation changes over time differed according to each site. Some CpGs were initially unmethylated (M value below −1) and became methylated (M value above 1) while other CpGs had M values above 1 that further increased over time (Figure 2B).

**Figure 3:**
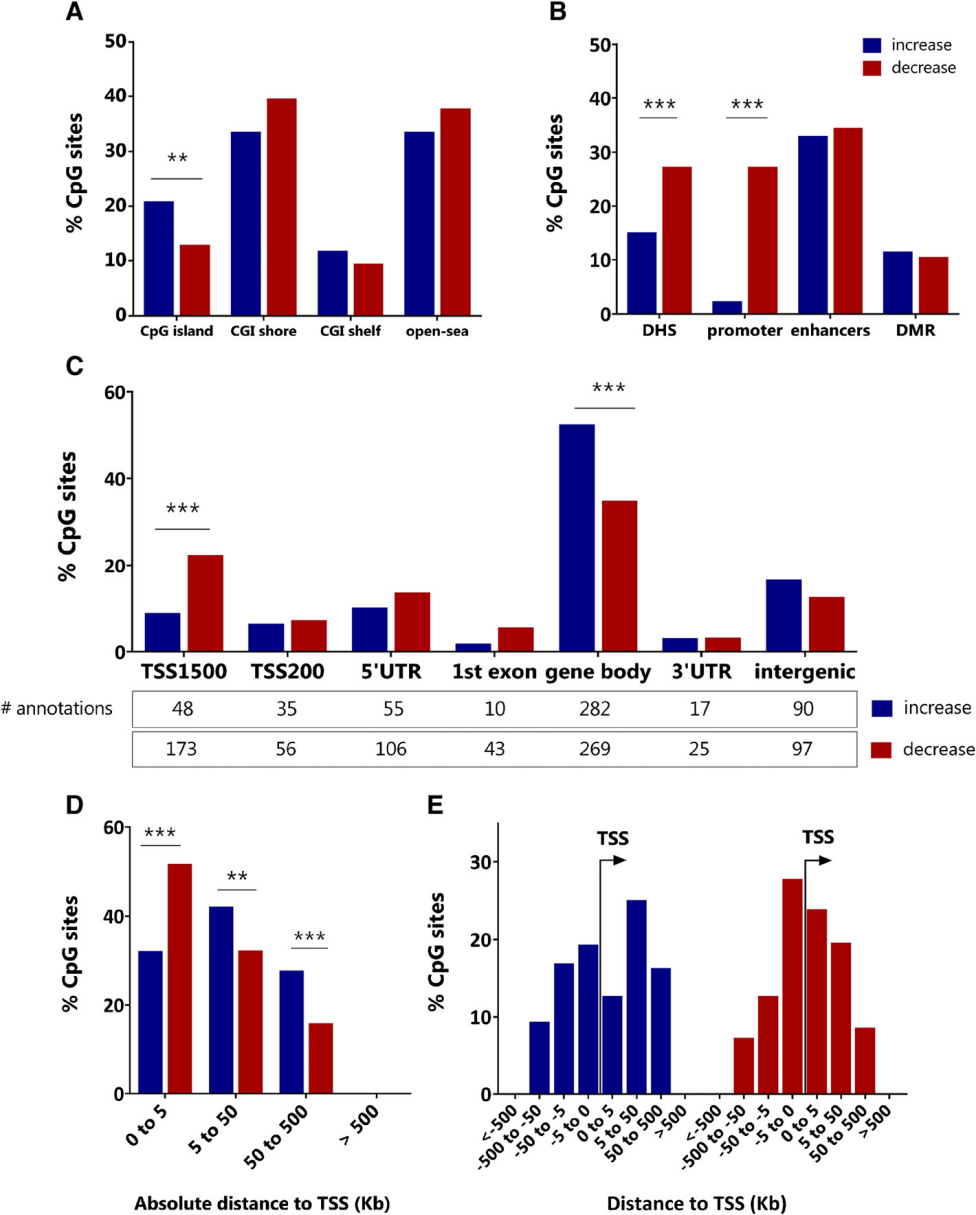
**Differences in the genomic distribution of age-modified CpG sites. (A)** Frequency of age-modified CpG sites according to the proximity to a CpG island (CGI). **(B)** Frequency of age-modified CpG sites according to regulatory annotations. **(C)** Frequency of age-modified CpG sites according to the gene location. TSS = transcriptional start site; UTR = untranslated region; age-methylated CpGs mapped to 537 gene locations and age-demethylated CpGs to 769 gene locations. **(D)** Frequency of age-modified CpG sites binned by absolute distance to the nearest TSS. **(E)** Frequency of age-modified CpG sites according to their location in relation to the nearest TSS (upstream/downstream).

Since the majority of age-modified CpG sites were associated to a known transcript (Figure 1D) and their location can provide insights on their putative biological relevance, we analysed the genomic distribution of the 794 age-modified CpG sites according to their proximity to a CpG island and other genomic regulatory features like DNAse I hypersensitivity sites (DHSs) and enhancers. The annotation to be inside a CpG island was significantly over-represented in age-methylated CpG sites (20.9%) compared to age-demethylated sites (12.9%) (χ^2^ = 8.44, *P* = 0.003), Figure 3A. There were no differences in the distribution of age-modified CpG sites with regard to CpG island shores (39.6% vs 33.6%, *P* = 0.08) or the ‘open sea’ (37.9% vs. 33.6%, *P* = 0.21) (Figure 3A). Regarding the connection of age-modified CpG sites with regulatory features, age-demethylated CpG sites were more frequently found in DHS (26.7% vs 14.5%, χ^2^ = 12.4, *P* = 0.0004) and promoter-associated regions (29.7% vs 3.3% χ^2^ = 88.2, *P* < 0.00001) than in age-methylated sites (Figure 3B). There were no differences in the distribution of age-modified CpG sites within enhancers or known differentially methylated regions (DMRs, Figure 3B).

### Differential TSS relationship between age-methylated and age-demethylated sites

We then investigated the distribution of age-modified CpG sites according to their position within the gene structure. Provided that any given CpG site can be annotated to a gene in more than one accession number (for instance, in case of isoforms or anti-sense transcripts), all locations associated to an age-modified CpG (TSS1500, TSS200, 5′UTR, 1st exon, gene body, 3′UTR and intergenic) were included in the analysis. We found that age-methylated CpG sites were over-represented in the gene body compared to age-demethylated CpG sites (52.5% vs 34.9%, χ^2^ = 39.8, *P* < 0.0001), and age-demethylated CpG sites were more frequently annotated within 1,500 bp of the transcriptional start site (TSS) compared to age-methylated sites (22.4% vs 8.93%, χ^2^ = 41.3, *P* < 0.0001), Figure 3C. To obtain further insights on their relationship with promoter regions, we calculated the position (upstream or downstream) and distance of each site to its nearest TSS. The distribution binned by the absolute distance revealed that about half of the age-demethylated CpG sites spanned within 0 to 5 kilobases (kb) of a TSS compared to age-methylated CpG sites (51.7% vs 32.1%, χ^2^ = 30.1, *P* = 0.0001). Conversely, age-methylated CpG sites were more frequently annotated from 5 to 50 kb of a TSS (42.1% vs 32.3%, χ^2^ = 7.0, *P* = 0.004) and from 50 to 500 kb (27.7% vs 15.9%, χ^2^ = 11.5, *P* = 0.0007), Figure 3D. We also found differences in the proportions regarding directionality to the TSS (upstream/downstream): age-demethylated sites were more frequent within −5 to +5 kb and age-methylated sites within +5 to +50 kb downstream of the TSS (Figure 3E).

**Figure 4:**
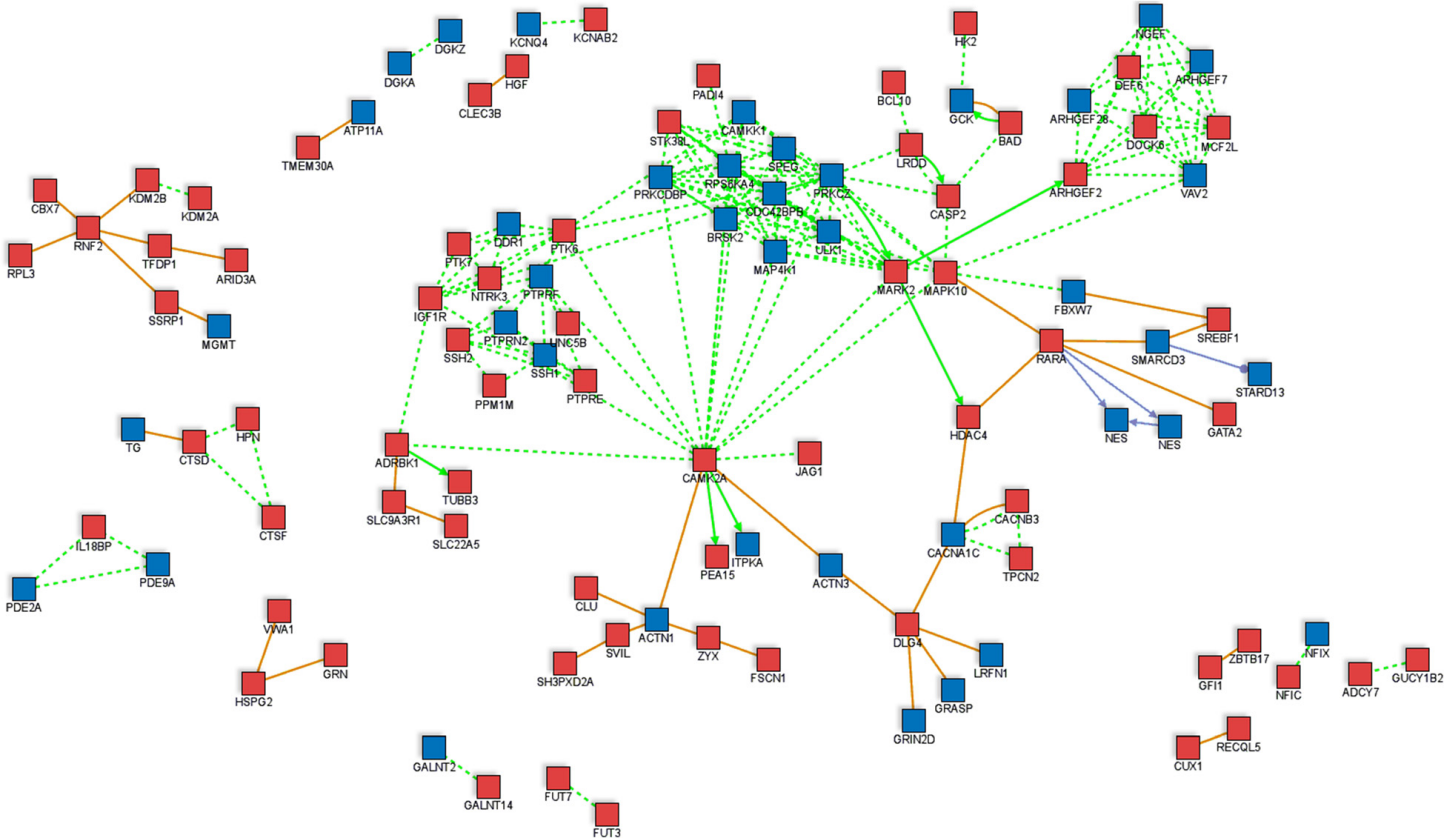
**Induced network analysis for the known protein-protein interactions between the products of genes containing age-modified CpG sites.** Genes harbouring age-modified CpG sites were used as seeds to identify known protein-protein interactions (orange line), connections in a biochemical reaction (solid and dotted green lines) and genetic regulation (purple line) at high level of confidence. Node colour represents if the gene is age methylated (blue) or age demethylated (red). The solid arrow in a biochemical reaction (green) indicates protein/substrate relationship. Non-connected seed nodes are not shown.

It is still a matter of debate whether age-associated changes in DNA methylation are biologically relevant. We evaluated which biological processes, cellular components and molecular functions were related to genes containing age-modified CpG sites (Additional file 3) and if there were known interactions between the age-modified loci. Induced network analysis using the combined list of age-methylated and age-demethylated loci revealed that several of these genes were known to interact within protein-protein complexes or biochemical reactions (Figure 4). The over-representation analyses were also performed with separated lists as an attempt to dichotomize relevant biological functions that might be specific to age-methylated and age-demethylated loci, and these results are explained below.

### Genes containing age-methylated CpG sites code for products involved in development, cell adhesion and the plasma membrane

**Figure 5:**
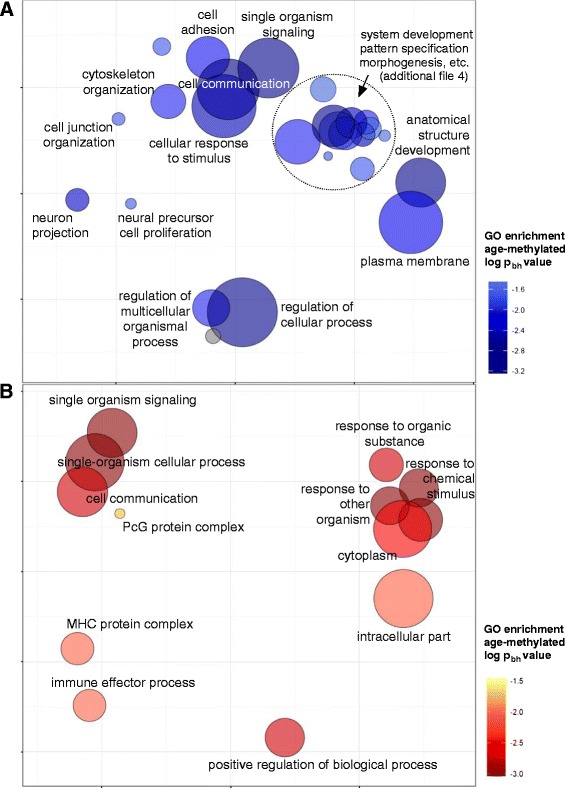
**Gene ontology (GO) categories significantly enriched in genes harbouring age-modified CpG sites.** Summary of GO categories presented in a two-dimensional space derived by applying multidimensional scaling to a pairwise distance matrix of the semantic similarities in GO terms. **(A)** Enriched GO categories in age-methylated CpG sites (blue); **(B)** Enriched GO categories in age-demethylated sites (red); colour scales represent the Benjamini-Hochberg corrected log *P* value for the enrichment (log *P* −2 equals *P* = 0.01). Circle sizes indicate the number of genes of each GO term (set size). Detailed information on enriched GO categories, number of age-modified loci per GO term and *P* values is presented in Additional file 3 (for age-methylated CpGs) and Additional file 4 (for age-demethylated CpGs). For this visualization approach, highly similar GO categories are grouped together and cluster representatives are selected based on *P* values and dispensability scores. Each GO term receives a coordinate so that more semantically similar GO terms get closer in the plot [58]. To be regarded as significant, any GO term requires coincidence of at least five genes and a p_bh_ = 0.05.

Gene ontology (GO) analysis revealed that age-methylated loci were significantly over-represented in the biological processes of development and morphogenesis of anatomical structures (Figure 5A and Additional file 4). We also found that genes having age-methylated CpGs were over-represented in neuronal-related functions (Figure 4A). The GO annotations of neuron part (GO:0097458, 20 genes), axon part (GO:0033267, seven genes) and neuron projection (GO:0043005, 17 genes) were the most significant in the enrichment based on cell components (Additional file 4). The over-representation of age-methylated loci within neuronal genes was also supported by the enrichment in the biological processes of transmission of nerve impulse (GO:0019226, 18 genes) and neural precursor cell proliferation (GO:0061351, five genes), Figure 5A and Additional file 4. Another two highly significant annotations for age-methylated loci included the plasma membrane (GO:0005886, 62 genes) and cell adhesion (GO:0007155, 20 genes), Figure 5A.

### Age-demethylated sites were enriched in GO categories of response to diverse stimuli, immune effector processes and the cytoplasm

**Figure 6:**
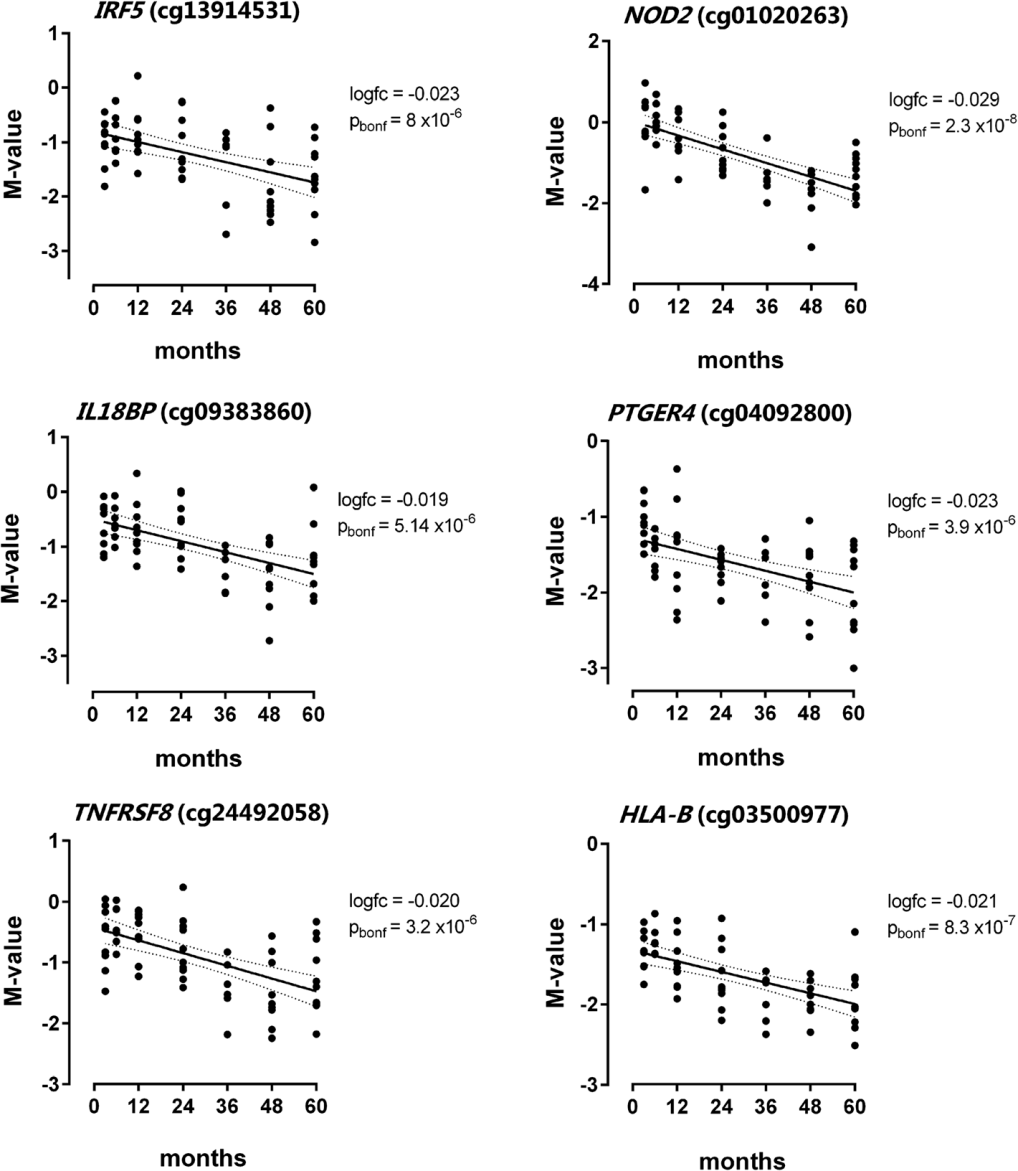
**Longitudinal trends of the DNA methylation levels in six immune genes within 3 to 60 months after birth.** DNA methylation levels are expressed as M value; each dot represents an individual. The dotted lines represent the 95% CI of the regression line; logfc = log fold change in methylation over time; p_bonf_ = Bonferroni-corrected *P* value. *IRF5* = interferon regulatory factor 5; *NOD2* = nucleotide-binding oligomerization domain containing 2; *IL18BP* = interleukin 18 binding protein; *PTGER4* = prostaglandin E receptor 4; *TNFRSF8* = tumour necrosis factor receptor superfamily, member 8; *HLA-B* = major histocompatibility complex, class I, B.

Genes containing age-demethylated CpG sites in blood leukocytes were significantly enriched in the biological processes of (1) response to diverse stimuli including microorganisms, chemicals and organic substances; (2) positive regulation of biological process; (3) immune effector process; and (4) cell communication and signalling, Figure 5B. Detailed information on the gene ontology enrichment for age-demethylated loci is presented in Additional file 5. Furthermore, genes harbouring age-demethylated sites were significantly enriched in the cellular components: cytoplasm (GO:00055737, 194 genes), intracellular-membrane-bound organelles (GO:0043231, 191 genes) and the Golgi apparatus (GO:0044431, 22 genes). Altogether, this indicates that demethylation in blood leukocytes within 3 to 60 months after birth is mainly related to the interaction of the cells with the environment and the development of immune effector responses. As shown in Figure 5B, we found that age-demethylated CpGs were enriched in genes of the major histocompatibility protein complex (MHC, chr. 6p21.3), including type I (*HLA-B*, *HLA-C*) and type II alleles (*HLA-DMA*, *HLA-DPB1*) as well as the MHC class I polypeptide-related sequence A (*MICA*). We also found age-demethylated loci in genes encoding defensins (*DEFA4*, *DEFB132*), prostaglandin receptors (*PTGER2*, *PTGER4*), members of the tumour necrosis factor superfamily (*TNFAIP8L1*, *TNFRSF8*, *TNFSF14*), interleukin 18 binding protein (*IL18BP*), interferon regulatory factor 5 (*IRF5*), leukotriene B4 receptor (*LTB4R*), the CD2 ligand on T cells (*CD58*) and pattern recognition receptors (*NOD2*). The longitudinal changes in DNA methylation levels for some CpG sites located in immune genes are presented in Figure 6. GO analysis also revealed that age-demethylated CpG sites were enriched in genes from the PcG protein complex (*CBX7*, *RNF2*, *KDM2B*, *JARID2*, *PHF1*), Figure 5B and Additional file 5.

### Age-modified CpG sites spanned over genes encoding chromatin remodelling factors and transcription factors

**Figure 7:**
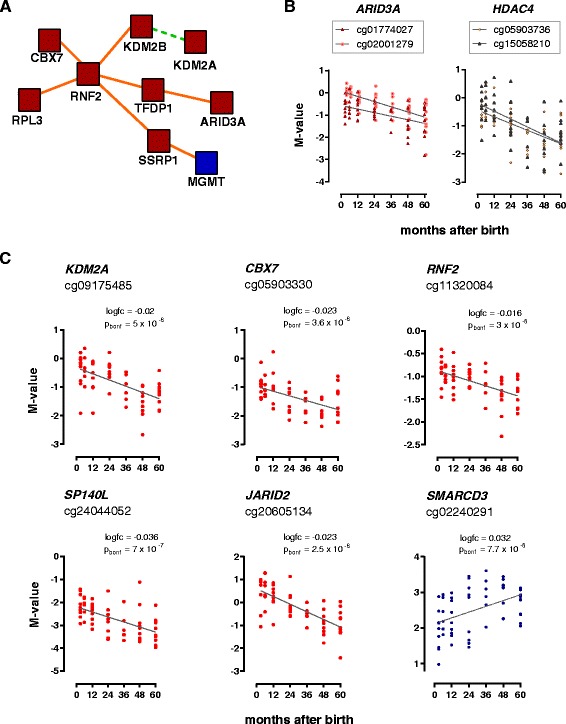
**DNA methylation levels within 3 to 60 months after birth in genes encoding histone modifiers and chromatin remodelers. (A)** Protein interactions among genes related to the chromatin remodelling machinery that contain age-modified CpG sites; protein-protein interaction (orange line); biochemical reaction (green line); factors encoded by age-demethylated genes (red) and age-methylated genes (blue). **(B)** Longitudinal changes in DNA methylation for two CpG sites in the genes encoding for AT-rich interactive domain-containing protein 3A (*ARID3A*) and the histone deacetylase 4 (*HDAC4*); each dot represents an individual. **(C)** Longitudinal changes in DNA methylation for six genes involved in the chromatin remodelling; each dot represents an individual. *KDM2A* = lysine (K)-specific demethylase 2A; *CBX7* = chromobox homolog 7; *RNF2* = E3 ubiquitin-protein ligase RING2; *SP140L* = SP140 nuclear body protein-like; *JARID2* = jumonji, AT-rich interactive domain 2; *SMARCD3* = SWI/SNF-related, matrix-associated, actin-dependent regulator of chromatin, subfamily d, member 3.

Together with the PcG complex, we found age-modified CpG sites in genes encoding histone modifiers and chromatin remodelling factors. These included the lysine-specific ‘K’ histone demethylases with F box domains (*KDM2A* and *KDM2B*), AT-rich interaction domains containing proteins (*JARID2* and *ARID3A*), the structure-specific recognition protein 1 (*SSRP1*), the SP140 nuclear body protein-like (*SP140L*) and the gene *SMARCD3* involved in the ATP-dependent chromatin remodelling complex (specific of neuronal progenitors). The known interactions for nine age-modified loci involved in chromatin remodelling are presented in Figure 7A. Some of these genes had more than one CpG site modified by age that followed the same trends of age-related changes (Figure 7B and Table 3). The DNA methylation changes over time in six genes annotated as chromatin/DNA binding proteins are presented in Figure 7C.

In addition, we found longitudinal changes in DNA methylation in several genes encoding transcription factors (TFs). A table with the annotation of the TF genes harbouring age-modified CpG sites is presented in Additional file 6. As expected, several CpG sites were found in TFs involved in development such as fork head boxes (*FOXI2*, *FOXK1* and *FOXK2*), T-boxes (*TBX1* and *TBX2*), ANTP/HOXL homeoboxes (*HOXA10*, *HOXA3*, *HOXB6*), the SRY-related HMG box (*SOX10*), ANTP/NKL homeoboxes (*VENTX*, *NKX2*) and CUT homeoboxes (*CUX1*). Several TFs involved in granulocyte differentiation, B-cell immunity and cytokine response were found containing age-modified CpG sites (Additional file 6). These include the nuclear factor of activated T-cell 4 (*NFATC4*), the interferon regulatory factor 5 (*IRF5*), the transcriptional regulator ERG (*ERG*), the nuclear hormone receptor *RARA* and the GATA zinc finger domain TF (*GATA2*). Induced network analysis using the list of genes having age-modified CpG sites revealed that several of these TF are known to interact with the proteins encoded by other age-modified genes as binary protein-protein interactions and/or biochemical reactions (Figure 4). With few exceptions, CpG sites that were age methylated in DIPP children were found methylated in adult blood, and CpG sites that were age demethylated in DIPP children were found demethylated in adult blood. A comparison of the DNA methylation levels (M values) between the children in this study and adult blood leukocytes is presented in Additional file 7.

## Discussion

Here we present a prospective analysis on the dynamics of DNA methylation in peripheral blood leukocytes during early childhood. Our study includes data on seven time points (from 3 to 60 months after birth) from the same ten individuals and reveals that DNA methylation levels are modified as a function of age in at least 794 CpG sites distributed in RNA coding genes as well as intergenic regions (Figure 1D). Several age-modified CpG sites are located within the same gene and spread in regions from few base pairs to kilobases (Tables 2 and 3). Our findings indicate that DNA methylation changes related to age may not only be due to stochastic DNA methylation drift [14,36] but rather correspond to a programme with functional relevance in leukocyte biology. We previously described a group of differentially methylated CpG signatures related to the lineage of sorted blood leukocytes in healthy adults [34]. In the present study, we found CpG methylation signatures that change as a function of age within the first 5 years after birth, independently of the individual. It is worth noting that some genes associated to chronic inflammatory diseases (for example, *NOD2*, *PTGER4*, *IRF5*, *ADAM33*) contain age-modified CpG sites in blood leukocytes.

**Figure 8:**
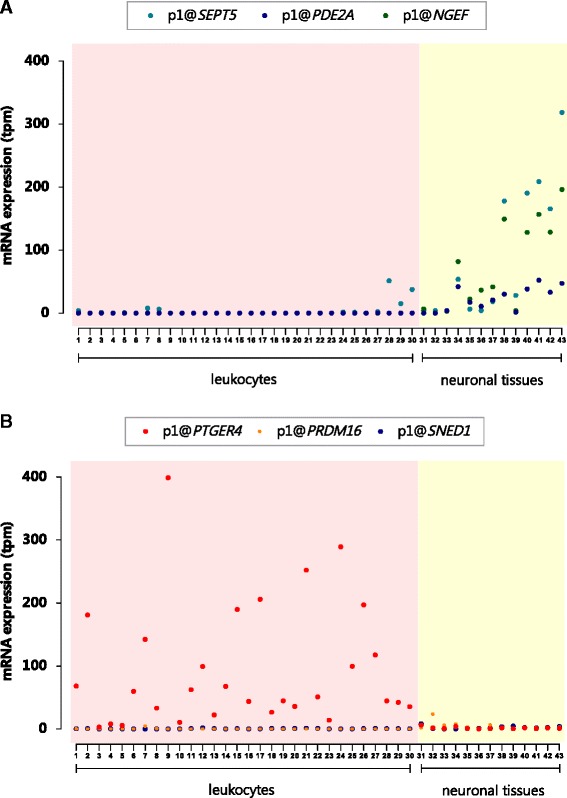
**mRNA levels of genes harbouring age-modified CpG sites based on the FANTOM5 consortium data. (A)** CAGE-defined TSS expression profiles for the age-methylated genes *NGEF*, *SEPT5* and *PDE2A* in purified primary leukocytes and brain tissues. **(B)** CAGE-defined TSS expression profiles for the age-demethylated genes *PTGER4* and *PRDM16* and the age-methylated gene *SNED1*; mRNA levels are presented in transcripts per million (TPM, *y*-axis). Forty-five samples from blood and neuronal lineages as evaluated by the FANTOM5 consortium [43] are represented in the *x*-axis. Detailed information on the samples included in this comparison is presented in Additional file 8.

Increased DNA methylation is involved in silencing developmental genes [37]. We found that genes with age-methylated CpGs are enriched in biological processes related to embryonic development and cell adhesion, as well as with the plasma membrane (Figure 5A and Additional file 4). Among the most important observations from this study is the differential genomic distribution of age-methylated CpG sites, which are more frequently located within 5 to 50 kb from the TSS and over-represented in gene bodies and intragenic CpG islands (Figure 3). This is very interesting because intragenic methylation can predict gene expression levels, it is crucial in regulating isoform splicing in neuronal genes [38] and it is over-represented in genes that guide the formation of junctions in the motor neurons [39]. We also found that CpG sites that are age methylated in blood leukocytes are commonly located in genes related to neuronal functions. Several of those (for example, *NEGF*, *SEPT5*, *PDE2A*,) show detectable mRNA expression in brain tissues but not in sorted blood leukocytes (Figure 8A). Besides, some genes related to immune functions were age methylated (for example, *IL17RD*) reflecting that in human leukocytes, differences in DNA methylation are tightly related with cell differentiation and commitment to lymphoid and myeloid lineages [40].

On the other hand, demethylation in promoter regions is known to facilitate gene expression [41]. Previous studies have shown that age-demethylated sites from birth to the first 2 years are enriched in immune-related genes [22]. Our results replicate these findings and also show that genes harbouring age-demethylated CpGs are enriched in genes related to the response to diverse stimuli including endogenous compounds and organic and chemical substances (Figure 5B and Additional file 5). Interestingly, age-demethylated CpGs were enriched in genes related to the cytoplasm, the intracellular organelles and the Golgi apparatus. These findings could in part be explained by demethylation of class I and class II MHC molecules as well as by demethylation of at least five enzymes involved in glycosylation pathways that are located in the Golgi apparatus (that is, *B3GALT4*, *GALNT14*, *ST6GAL2*, *FUT7* and *FUT3*). Moreover, we identified CpG sites in genes encoding histone modifiers and chromatin remodelling factors that become demethylated in blood leukocytes by increasing age. The implicated molecules have histone demethylase activity (*JARID2*, *KDM2A* and *KDM2B*) and histone deacetylase activity (*HDAC4*, *NACC2*) (Figure 7). The demethylation of genes encoding histone demethylases may contribute to the dynamic changes that occur in blood leukocytes during this period of life and may facilitate their maturation towards subpopulations. For instance, global DNA methylation remodelling has been observed in the transition from naïve to memory T cells [42]. In this sense, age-modified loci may participate as functional intermediates in a cascade of events that contribute to leukocyte maturation. Connections to the epigenetic machinery are further suggested by the identification of five age-modified CpG sites in genes encoding microRNAs: three age-methylated sites in *MIR219-2, MIR183/MIR96* and *MIRLET7A3*/*MIRLET7B* and two age-demethylated sites in *MIR10A* and *MIR574* (Additional file 1)*.*

More studies are needed to investigate which mechanisms direct the methylation machinery to these age-modified loci during this time window; and also to elucidate the connection between age-demethylated loci and mRNA expression in blood leukocytes. This study revealed that age-demethylated CpG sites are more frequently located in DHS, in promoters and in close proximity to the TSS (Figure 3), suggesting that these changes in methylation may be biologically relevant at the transcriptional level. We found significant GO categories related to the immune system, and using the FANTOM5 data [43], we observed that some age-demethylated genes are indeed expressed in peripheral blood leukocytes but not in other tissues (for example, *PTGER4*, Figure 8B and Additional file 8). In agreement with previous studies showing that age-induced differential methylation may occur without changes in gene expression [44], we found genes with DNA methylation changes over time but without detectable differences in expression (Figure 8B and Additional file 8). Further studies are needed to elucidate which proportion of the age-associated changes in DNA methylation are part of a ‘programme’, how many are stochastic, which ones contribute to differential gene expression and how many are tissue independent or tissue specific.

Previous studies have found age-modified CpG sites that are restricted to certain tissues [45]. However, age-modified CpG sites have been detected in tissues that originate from distinct germ layers, suggesting that tissue-independent changes do occur. For instance, a common age-modified methylation module has been found in whole blood and brain tissue [46]; others have described common age-modified signatures within the whole blood, lung tissue and cervix [27], and studies in adult women revealed age-modified CpG sites in the blood that showed concordant patterns in other non-haematopoietic tissues [7]. Among the reported epigenetic biomarkers of ageing in adult’s samples, we validated one age-demethylated CpG site in *FHL2* (cg06320277, p_bonf_ = 8.44 × 10^−6^) but did not detect significant differences for other reported age biomarkers [11,12], suggesting that age-modified loci may differ between children and adults. We also found concordance with 34 age-modified CpG sites that were previously described by Alisch *et al*., in peripheral blood leukocytes in paediatric populations [20], and 11 differentially methylated CpG sites described by Martino *et al*., comparing mononuclear cells from cord blood and children age 1 year [22]. Common loci between ours and these studies included *TSPO*, *GAL3ST1*, *BST2*, *ASB16*, *MARK2* and the inner-ear expressed genes *OTOS* (otospiralin) and *TMC2*. These common age-modified loci were identified in studies conducted in males [23] and females [22].

Provided that we filtered out cell-type-specific CpG sites from the list of age-modified CpGs and some of the age-modified CpG sites have been previously detected by using fractionated and unfractionated blood, it is less likely that compositional differences in cell counts may have affected these observations. Additional insights about common, non-tissue-specific, age-related methylation signatures were obtained from the identification of 29 CpG sites that were age modified in this study and also found differentially methylated in the buccal epithelium of twins between birth and the age of 18 months [21]. These sites mapped to 21 know genes including *ARID3A*, *KLF9*, *NOD2*, *PRKCZ*, *SOX10*, *SPEG*, *TEPP*, *TRIM7*, *TTC22* and *ZNF710*. The gene *ARID3A* is very interesting because it was found containing four age-demethylated CpG sites in a region of 6.98 kb. This molecule is expressed in leukocytes of myeloid origin and is involved in normal embryogenesis and haematopoiesis. Observed age effects on the DNA methylation levels of *ARID3A* within the first 2 years of life have also been reported in children with a different genetic background and environmental setting [23], as well as in males [20]. Furthermore, the identification of age-modified CpG sites in several genes related to the formation of organs from the three germinal layers (Additional file 4) suggests that for some loci, the peripheral blood leukocytes remember an age-related programme that is common across different tissues. The results of this study suggest the existence of age-modified loci that are not leukocyte specific but can be detected in blood as a surrogate tissue.

To our knowledge, this is the first time the same individuals have been followed for this number of time points at this early age rendering 60 samples for analysis. The number of age-modified CpGs detected in this study (*n* = 794) is lower compared to those previously described, reflecting a very stringent statistical model that calculated the variation over many time points and included the individual as covariate. Several factors (gender, lifestyle, environmental exposures, sequence variants *in cis*,) may influence the dynamics in which a given CpG site is methylated or demethylated during lifetime. We could not rule out that environmental differences like season of birth, maternal smoking, breastfeeding, mode of delivery, infections and/or vaccinations may have introduced sources of variation [47,48]. Nevertheless, we included the parameter related to the individuals in order to attenuate the possible confounding effect coming from the repeated sampling procedure. We think that in combination with assuming additive (and close to linear effects), the model applied here reduced the list of age-modified CpGs to those that have less interindividual variability, some even previously observed. Assuming an additive model in this sense is probably suboptimal but reasonably effective to remove very strong individual’s related effects. It should be mentioned that other analytical strategies such as mixed effects models, which allows a random intercept by individual, are suitable for this type of longitudinal analysis; however, we did not use this approach in this specific study because mixed models with such a big number of probes is computationally expensive and might suffer from the fact that each probe might respond differently from the others.

Another serious limitation of this study is that we measured DNA methylation in unfractionated blood and did not have differential cell counts at the time of sampling to adjust the analysis. In an attempt to remove as much as possible the confounding effects due to differential cell composition, we filtered the list of age-modified CpG sites against those identified as cell-type specific for leukocyte populations. We are aware that filtering age-modified CpG sites in children by the locations having differential methylation in sorted leukocytes in adults is suboptimal, but it is still the best that can be done to date; however, we believe that not considering the locations showing differential methylation in adulthood is not detrimental for this analysis and is still beneficial as it allows focusing on functionally relevant features. On the other hand, using existing methods for data deconvolution based on the adult cell-specific methylation profiles is risky as this data might not be relevant in children samples with a physiologically different cell composition and, hence, it might produce artefacts. Further studies are needed to address this point properly. A larger prospective study on longitudinal changes in DNA methylation during childhood is now ongoing in our laboratory including both males and females exposed to different lifestyles.

## Conclusions

This study provides a catalogue of 794 age-modified CpG sites that robustly reflect the changes in DNA methylation levels that occur in human blood leukocytes within 3 to 60 months after birth. Age-methylated CpG sites are significantly over-represented in genes involved in developmental and neuronal-related functions indicating that DNA methylation might play an important role in regulating differentiation and leukocyte-specific functions. On the other hand, genes harbouring age-demethylated sites reflect not only the immunological window in childhood but also suggest that blood leukocytes undergo a programme that allows their interaction with environmental factors and genome remodelling. The fact that methylation in several genes implicated in the physiopathology of inflammatory diseases is modified during the first years of life opens new perspectives on the role of environmental exposures and strategies for primary prevention. Our results provide valuable information on age-modified loci that can be useful for developing tools to correct for age effects when performing DNA methylation studies in children.

## Methods

### Study population

Ten healthy girls were selected from the Type 1 Diabetes Prediction and Prevention Study (DIPP) [49] to conduct a prospective genome-wide methylation analysis during childhood. The children were selected based on the availability of prospective samples, and that all remained healthy and seronegative for the T1D-associated antibodies (ICA, IAA, GADA and IA-2A) by 10 years of age. The DIPP study was launched in 1994 in Finland as a genetic screening programme for type 1 diabetes (T1D) risk alleles in newborn infants from the general population. The children included in this study were born between March 2000 and November 2002 in Tampere, Finland; all followed the Finnish vaccination programme and were carriers of the HLA-DQB1*03:02 allele but lacking DQB1*06:02 allele. The HLA-DR-DQ genotypes of the children as well as genotype-associated risk classes [50] are presented together with demographical characteristics in Table 1. Blood samples were collected during visits to the study centre at 3, 6, 12, 24, 36, 48 and 60 months after birth. Information on the clinical history of autoimmune diseases and exposures to diverse environmental factors (infections, diet, domicile, living habits, vaccinations,) was also collected. This study was conducted in accordance with the ethical principles for medical research stated in the Helsinki Declaration. The ethical committee of the Tampere University Hospital (Tampere, Finland) approved this study. Written informed consent was obtained from the parents of all the participants.

### Blood samples

Blood samples were taken in sodium citrate tubes and processed within 1 h from venipuncture. Samples were centrifuged at 1,700 g during 10 min at room temperature. After plasma collection, the buffy coat layer was removed to a separate the cryotube and contaminated red blood cells were lysed using osmotic shock in sterile water. The buffy coat containing unfractionated leukocytes was then pelleted by centrifugation, supernatant was removed and cells were suspended in sterile water and pipetted to a separate cryotube. Samples were stored at −80°C until DNA extraction.

### DNA extraction and DNA methylation measurements

Genomic DNA from peripheral leukocytes was extracted from buffy coats using the FlexiGene kit (QIAGEN, Hilden, Germany, Cat # 51204). DNA samples (*n* = 70) were diluted at 100 ng/μl in TE buffer (pH 8.0). The mean value for the A260/280 coefficient was 1.90 ± 0.05. DNA samples were diluted at 11 ng/μl, randomized in a 96-well plate and bisulfite treated using the EZ-96 DNA Methylation™ Kit (ZYMO Research, Irvine, CA, USA, Cat # D5004) according to the manufacturer’s instructions. Six DNA samples with 0%, 50% and 100% methylation (two of each) were included as controls (EpiTect Control DNA, QIAGEN, Cat # 59665 and Cat # 59655). Nine technical duplicates of the study samples were included to evaluate inter-assay correlations. Denatured bisulfite-treated DNA was amplified, fragmented and hybridized onto the HumanMethylation450 BeadChip (Illumina, Cat # WG-314-1003) following manufacturer instructions at the Bioinformatics and Expression Core Facility (BEA, Karolinska Institutet, Stockholm, Sweden). After extension and staining steps, the chips were scanned using the Illumina iScan (Illumina, San Diego). The Infinium methylation data are available in the Gene Expression Omnibus (GEO) database under the accession number GSE62219.

### Quality control and data normalization

Image analysis and signal detection were done using the Genome Studio Software. The quality control (QC) included the evaluation of detection *P* values, staining, extension, hybridization, bisulfite conversion and specificity. The *lumi* package was then used for pre-processing and normalization of the data [51]. The QC also included unsupervised hierarchical clustering and principal component analysis (PCA) on sample relationships based on CpG sites. The data was processed exactly as described previously [34] and QC verified as raw data and also after normalization by the quantile method. Based on these analyses, 60 biological samples passed QC and were studied (Table 1). Methylation levels in the 0%, 50% and 100% controls resulted as expected.

### Statistical analysis on differential methylation

DNA methylation levels were log_2_ transformed to M values and then statistically evaluated using the *limma* package [33]. A single procedure consisting of two steps was used to infer the association between age and DNA methylation, which resulted in a unique list of differentially methylated CpG sites. First, a linear model was used considering the age and the individual (repeated samples from the same person); the study of the variance was performed at this step, but no list of differentially methylated probes was generated. The information on the variance was then utilized as prior for the second step of the analysis, which consisted of a moderated *t*-test to compare the samples between the earliest and the latest time points (that is, 3 months vs 60 months after birth). The magnitude of the change in M values over time is indicated by the logfc: negative values indicate how much a CpG site decreases in methylation with age, while positive values indicate how much a CpG site increases in methylation. The moderated *t*-statistic is expressed as the column *t*. The significance level was set at *P* = 0.01 after multiple testing correction according to the Bonferroni method (p_bonf_).

### Data filtering of differentially methylated CpG sites

Fifty nine of the age-modified CpG sites had a single nucleotide polymorphism (SNP) annotated within less than ten base pairs (bp) from the query site and 99 CpG sites with a SNP annotated within the probe but >10 bp of the query site. The minor allele frequency (MAF) of each SNP within the probe sequence was interrogated in the Finnish population using ENGINES (Entire Genome Interface for Exploring SNPs) [52], and CpG sites containing a SNP in the probe with MAF above 0.01 were filtered out (*n* = 48). Furthermore, to avoid the confounding effects of CpG sites that are differentially methylated among leukocyte populations (cell-type specific), all age-modified CpG sites were contrasted against a list of 2,228 CpG sites with significant differential DNA methylation in sorted leukocytes [34] that serve as cell-type classifiers. Eleven age-modified CpG sites were found annotated as having significant DNA methylation differences within sorted leukocytes and therefore excluded. Given that all individuals were females, we did not filter out probes based on cross-hybridization [53].

### Genomic distribution and annotation of the features

The distribution of age-modified CpG sites according to their relation to a CpG island, gene structure or regulatory functions (DNAse I hypersensitivity site, promoter, enhancer or known DMR) was calculated based on the UCSC Genome Browser annotations provided by Illumina. To calculate statistics on the location of age-modified CpG sites (TSS1500, TSS200, 5′UTR, 1st exon, gene body, 3′UTR and intergenic), we included all the annotations connected to a site. The distance of any given CpG site to the nearest TSS was calculated by PeakAnalyzer [54]. The absolute distance and position in relation to single nearest TSS within 1,000 kb was calculated by the Genomic Regions Enrichment of Annotations Tool [55]. The comparisons on the frequency of age-modified CpG sites (age-methylated vs age-demethylated) according to their relation to CpG islands, gene structure or regulatory features (present: yes/no) were performed by using χ^2^ and Fisher’s exact test. A *P* < 0.05 was considered statistically significant.

### Enrichment analyses

Gene ontology analyses were conducted using the DAVID Bioinformatic Resource tool (v 6.7), ConsensusPathDB [56] and WebGesalt (WEB-based GEne SeT AnaLysis Toolkit) [57]. Enrichment significance was determined using the hypergeometric distribution and considered significant if at least five genes of the input list coincide with the gene set of a given gene ontology (GO) category, with a nominal *P* value <0.01 and Benjamini-Hochberg *P* value <0.05 (p_bh_). Visualization of enriched gene ontology terms was done by REVIGO based on semantic similarity-based scatterplots [58]. Annotations on gene families were obtained from PANTHER [59]. Induced network analyses were conducted by ConsensusPathDB to visualize known interactions between the protein products of the genes harbouring age-modified loci [56].

## Additional files

Additional file 1:
**A catalogue of the age-modified CpG sites identified in this study.**
Additional file 2:
**Gene families containing age-modified CpG sites.**
Additional file 3:
**Gene ontology (GO) analysis using the combined list of genes containing age-methylated and age-demethylated CpG sites.**
Additional file 4:
**Gene ontology (GO) categories significantly enriched in genes containing age-methylated sites.**
Additional file 5:
**Gene ontology (GO) categories significantly enriched in genes containing age-demethylated sites.**
Additional file 6:
**Transcription factor genes harbouring age-modified CpG sites within 3 to 60 months after birth in blood leukocytes.**
Additional file 7:
**DNA methylation levels in age-modified CpGs located on transcription factors and chromatin binding proteins.** The histogram represents the M value: blue (methylated) and red (demethylated). A comparison with adult blood (WB) and sorted leukocytes as described by [34] is included for comparative purposes. PBMC = peripheral blood mononuclear cells. Each individual girl is represented by a column from 3 months (left) to 60 months (right). Additional file 8:
**Gene expression levels for five genes containing age-associated CpG sites across 43 tissues from the FANTOM5 project** [43].

## Abbreviations

ARID3A: AT-rich interactive domain-containing protein 3A
CAGE: Cap-analysis of gene expression
CGI: CpG island
DIPP: Type 1 Diabetes Prediction and Prevention Study
GO: gene ontology
HDAC4: histone deacetylase 4
HLA: human leukocyte antigen
IRF5: interferon regulatory factor 5
JARID2: Jumonji, AT-rich interactive domain 2
KDM2A: lysine (K)-specific demethylase 2B
KDM2B: lysine (K)-specific demethylase 2B
MAF: minor allele frequency
MHC: major histocompatibility complex
NOD2: nucleotide-binding oligomerization domain containing 2
P_bonf_: *P* value adjusted by Bonferroni correction
P_bh_: *P* value adjusted by Benjamini-Hochberg
PcG: polycomb group
PTGER4: prostaglandin E receptor 4
SMARCD3: SWI/SNF-related, matrix-associated, actin-dependent regulator of chromatin, subfamily D, member 3
SNP: single nucleotide polymorphism
TF: transcription factor
TSS: transcription start sites
