# Supplementary material for: Age-associated DNA methylation changes in immune genes, histone modifiers and chromatin remodeling factors within 5 years after birth in human blood leukocytes
Source: Clin Epigenetics. 2015 Mar 26;7(1):34. doi: 10.1186/s13148-015-0064-6 (PMC4396570; doi:10.1186/s13148-015-0064-6)
Supplement: Additional file 2: — Gene families containing age-modified CpG sites. [file 13148_2015_64_MOESM2_ESM.docx]

| Additional file 2. Gene families containing age-modified CpG sites | | | |
| --- | --- | --- | --- |
| **Gene family** | **Gene symbol** | **Locus** | **Age-effect on methylation** |
| Actinins | *ACTN1* | 14q24 | 🡩 |
|  | *ACTN3* | 11q13.1 | 🡩 |
| A disintegrin and metalloproteinase (ADAM) | *ADAM33* | 20p13 | 🡩 |
|  | *ADAMTS2* | 5qter | 🡩 |
| Rho GTPase activating proteins | *ARHGAP17* | 16p12.1 | 🡫 |
|  | *ARHGEF2* | 1q21-q22 | 🡫 |
|  | *ARHGEF7* | 13q34 | 🡩 |
| beta-1,3-galactosyltransferase (beta3GalT) | *B3GALT4* | 6p21.3 | 🡫 |
|  | *B4GALNT1* | 12q13.3 | 🡩 |
| claudins | *CLDN2* | Xq22.3-q23 | 🡫 |
|  | *CLDN5* | 22q11.21 | 🡩 |
| C-type lectin domain | *CLEC16A* | 16p13.13 | 🡩 |
|  | *CLEC3B* | 3p22-p21.3 | 🡫 |
| Prostaglandin receptors | *PTGER2* | 14q22 | 🡫 |
|  | *PTGER4* | 5p13.1 | 🡫 |
| Solute carrier family | *SLC1A5* | 19q13.3 | 🡫 |
|  | *SLC22A18;*  *SLC22A18AS* | 11p15.5 | 🡫 |
|  | *SLC22A5* | 5q23.3 | 🡫 |
|  | *SLC22A8* | 11q11 | 🡫 |
|  | *SLC35F2* | 11q22.3 | 🡫 |
|  | *SLC35F3* | 1q42.2 | 🡩 |
|  | *SLC43A2* | 17p13.3 | 🡫 |
|  | *SLC9A3R1* | 17q25.1 | 🡫 |
|  | *SLCO3A1* | 15q26 | 🡩 |
| tumor necrosis factor | *TNFAIP8L1* | 19p13.3 | 🡫 |
|  | *TNFRSF8* | 1p36 | 🡫/🡩 |
|  | *TNFSF14;TNFSF14* | 19p13.3 | 🡫 |
| tripartite motif family | *TRIM7* | 5q35.3 | 🡩 |
|  | *TRIM15* | 6p21.3 | 🡩 |
|  | *TRIM26* | 6p21.3 | 🡫 |
|  | *TRIM50* | 7q11.23 | 🡩 |

Chr. = chromosome

🡩 = increase in methylation

🡫 = decrease in methylation
