# Supplementary material for: Age-associated DNA methylation changes in immune genes, histone modifiers and chromatin remodeling factors within 5 years after birth in human blood leukocytes
Source: Clin Epigenetics. 2015 Mar 26;7(1):34. doi: 10.1186/s13148-015-0064-6 (PMC4396570; doi:10.1186/s13148-015-0064-6)
Supplement: Additional file 4: — Gene ontology (GO) categories significantly enriched in genes containing age-methylated sites. [file 13148_2015_64_MOESM4_ESM.docx]

| Additional file 4. Gene ontology (GO) categories significantly enriched in genes containing age-methylated sites | | | | | |
| --- | --- | --- | --- | --- | --- |
| ***Biological process*** |  |  |  |  |  |
| **Category name** | **GO ID (level)** | **Set size** | **# age-modified genes (%)** | **nominal**  **P-value*** | **BH-adj**  **P value**** |
| single-multicellular organism process | GO:0044707 (2) | 5790 | 88 (1.5) | 1.08 x 10^-7^ | 7.46 x 10^-6^ |
| anatomical structure morphogenesis | GO:0009653 (2) | 2167 | 41 (1.9) | 9.94 x 10^-6^ | 0.0003 |
| anatomical structure development | GO:0048856 (2) | 4266 | 64 (1.5) | 2.65 x 10^-5^ | 0.0006 |
| *system development* | GO:0048731 (3) | 3633 | 57 (1.6) | 3.42 x 10^-5^ | 0.002 |
| *nervous system development* | GO:0007399 (4) | 1815 | 35 (1.9) | 3.48 x 10^-5^ | 0.01 |
| *pattern specification process* | GO:0007389 (3) | 440 | 14 (3.2) | 8.15 x 10^-5^ | 0.004 |
| *multicellular organismal development* | GO:0007275 (3) | 4211 | 62 (1.5) | 9.3 x 10^-5^ | 0.004 |
| *embryo development* | GO:0009790 (3) | 976 | 22 (2.3) | 0.0001 | 0.005 |
| *sensory organ development* | GO:0007423 (3) | 457 | 13 (2.8) | 0.0004 | 0.01 |
| *cellular component morphogenesis* | GO:0032989 (3) | 1072 | 22 (2.1) | 0.0005 | 0.01 |
| *cytoskeleton organization* | GO:0007010 (3) | 867 | 19 (2.2) | 0.0005 | 0.01 |
| *organ development* | GO:0048513 (3) | 2583 | 40 (1.6) | 0.0009 | 0.016 |
| *tube development* | GO:0035295 (3) | 462 | 12 (2.6) | 0.001 | 0.02 |
| *hearth development* | GO:0007507 (3) | 415 | 10 (2.4) | 0.006 | 0.03 |
| *digestive tract development* | GO:0048565 (3) | 117 | 5 (4.3) | 0.005 | 0.03 |
| *muscle structure development* | GO:0061061 (3) | 501 | 12 (2.4) | 0.003 | 0.02 |
| regulation of cellular process | GO:0050794 (3) | 8136 | 105 (1.3) | 1.15 x 10^-5^ | 0.002 |
| cell communication | GO:0007154 (3) | 5068 | 73 (1.4) | 2.63 x 10^-5^ | 0.002 |
| single organism signaling | GO:0044700 (2) | 4949 | 71 (1.4) | 4.29 x 10^-5^ | 0.0007 |
| single organism developmental process | GO:0044767 (2) | 4750 | 67 (1.4) | 0.0001 | 0.001 |
| cellular response to stimulus | GO:0051716 (2) | 5416 | 72 (1.3) | 0.0004 | 0.004 |
| multicellular organisms signaling | GO:0035637 (3) | 800 | 18 (2.2) | 0.0006 | 0.01 |
| regulation of multicellular organism process | GO:0051239 (3) | 2022 | 34 (1.7) | 0.0006 | 0.01 |
| cell adhesion | GO:0007155 (2) | 996 | 20 (2.0) | 0.001 | 0.009 |
| *cell-substrate adhesion* | GO:0031589 (3) | 230 | 8 (3.5) | 0.001 | 0.02 |
| *cell junction organization* | GO:0034330 (3) | 213 | 7 (3.3) | 0.004 | 0.02 |
| actin filament-based process | GO:0030029 (3) | 519 | 12 (2.3) | 0.004 | 0.02 |
| ensheathment of neurons | GO:0007272 (3) | 90 | 5 (5.6) | 0.001 | 0.02 |
| neural precursor cell proliferation | GO:0061351 (3) | 103 | 5 (4.9) | 0.003 | 0.02 |
| transmission of nerve impulse | GO:0019226 (4) | 774 | 18 (2.3) | 0.0004 | 0.03 |
| ***Cellular components*** |  |  |  |  |  |
| **Category name** | **GO ID (level)** | **Set size** | **# age modified genes (%)** | **nominal**  **P-value*** | **BH-adj**  **P value**** |
| neuron part | GO:0097458 (2) | 803 | 20 (2.5) | 7.72 x 10^-5^ | 0.002 |
| *axon part* | GO:0033267 (3) | 122 | 7 (5.7) | 0.0001 | 0.005 |
| *neuron projection* | GO:0043005 (3) | 652 | 17 (2.6) | 0.0001 | 0.005 |
| *neuronal cell body* | GO:0043025 (3) | 279 | 9 (3.2) | 0.001 | 0.02 |
| plasma membrane | GO:0005886 (2) | 4517 | 62 (1.4) | 0.0006 | 0.008 |
| *cell cortex* | GO:0005938 (4) | 208 | 8 (3.8) | 0.0008 | 0.03 |
| adherens junction | GO:0005912 (3) | 206 | 7 (3.4) | 0.003 | 0.05 |
| *Calculated according to the hyper-geometric test  **BH=Benjamini-Hochberg P value | | | | | |
