# Supplementary material for: Age-associated DNA methylation changes in immune genes, histone modifiers and chromatin remodeling factors within 5 years after birth in human blood leukocytes
Source: Clin Epigenetics. 2015 Mar 26;7(1):34. doi: 10.1186/s13148-015-0064-6 (PMC4396570; doi:10.1186/s13148-015-0064-6)
Supplement: Additional file 5: — Gene ontology (GO) categories significantly enriched in genes containing age-demethylated sites. [file 13148_2015_64_MOESM5_ESM.docx]

| Additional file 4. Gene ontology (GO) categories significantly enriched in genes containing age-demethylated sites | | | | | |
| --- | --- | --- | --- | --- | --- |
| ***Biological process*** |  |  |  |  |  |
| **Category name** | **GO ID (level)** | **Set size** | **# age-modified genes (%)** | **nominal**  **P-value*** | **BH-adj**  **P value**** |
| cellular response to stimulus | GO:0051716 (2) | 5416 | 127 (2.4) | 3.92 x 10^-6^ | 0.0001 |
| response to biotic stimulus | GO:0009607 (2) | 677 | 26 (3.9) | 6.37 x 10^-5^ | 0.001 |
| single-organism cellular process | GO:0044763 (2) | 10841 | 214 (2.0) | 4.21 x 10^-5^ | 0.001 |
| single organism signaling | GO:0044700 (2) | 4949 | 113 (2.3) | 7.16 x 10^-5^ | 0.001 |
| *signal transduction* | GO:0007165 (4) | 4424 | 108 (2.5) | 5.5 x 10^-6^ | 0.002 |
| response to chemical stimulus | GO:0042221 (2) | 3063 | 77 (2.5) | 9.08 x 10^-5^ | 0.001 |
| response to other organism | GO:0051707 (2) | 647 | 25 (3.9) | 7.95 x 10^-5^ | 0.001 |
| *modification of morphology or physiology of other organism* | GO:0035821 (2) | 396 | 14 (3.5) | 0.007 | 0.04 |
| positive regulation of biological process | GO:0048518 (3) | 3785 | 93 (2.5) | 3.01 x 10^-5^ | 0.002 |
| *positive regulation of cellular process* | GO:0048522 (4) | 3350 | 83 (2.5) | 7.4 x 10^-5^ | 0.01 |
| response to organic substance | GO:0010033 (3) | 2137 | 60 (2.8) | 3.19 x 10^-5^ | 0.002 |
| *cellular response to organic substance* | GO:0071310 (4) | 1586 | 44 (2.8) | 0.0005 | 0.04 |
| cell communication | GO:0007154 (3) | 5068 | 117 (2.3) | 2.85 x 10^-5^ | 0.002 |
| regulation of response to stimulus | GO:0048583 (3) | 2681 | 68 (2.5) | 0.0002 | 0.01 |
| immune effector process | GO:0002252 (2) | 582 | 20 (3.5) | 0.001 | 0.01 |
| *antigen processing and presentation* | GO:0019882 (2) | 219 | 10 (4.6) | 0.003 | 0.02 |
| *immune response* | GO:0006955 (2) | 1317 | 34 (2.6) | 0.007 | 0.04 |
| ***Cellular components*** |  |  |  |  |  |
| **Category name** | **GO ID (level)** | **Set size** | **# age-modified genes (%)** | **nominal**  **P-value*** | **BH-adj**  **P value** |
| cytoplasm | GO:0005737 (3) | 9599 | 194 (2.0) | 4.4 x 10^-5^ | 0.003 |
| intracellular part | GO:0044424 (2) | 12746 | 239 (1.9) | 0.0002 | 0.009 |
| *PcG protein complex* | GO:0031519 (3) | 40 | 5 (12.5) | 0.0005 | 0.02 |
| *MHC protein complex* | GO:0042611 (3) | 38 | 5 (13.1) | 0.0004 | 0.01 |
| intracellular membrane-bound organelle | GO:0043231 (3) | 9587 | 191 (1.9) | 0.002 | 0.03 |
| Golgi apparatus part | GO:0044431 (3) | 672 | 22 (3.2) | 0.003 | 0.04^#^ |
| *Calculated according to the hypergeometric test  **BH=Benjamini-Hochberg P value  MHC: Major histocompatibility complex  PcG: Polycomb group  ^#^enrichment statistics from WebGesalt | | | | | |
