# Supplementary figures and images for: Age-associated DNA methylation changes in immune genes, histone modifiers and chromatin remodeling factors within 5 years after birth in human blood leukocytes
Source: Clin Epigenetics. 2015 Mar 26;7(1):34. doi: 10.1186/s13148-015-0064-6 (PMC4396570; doi:10.1186/s13148-015-0064-6)

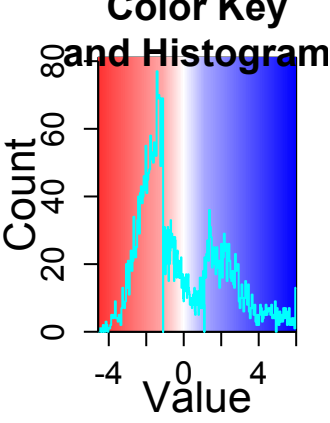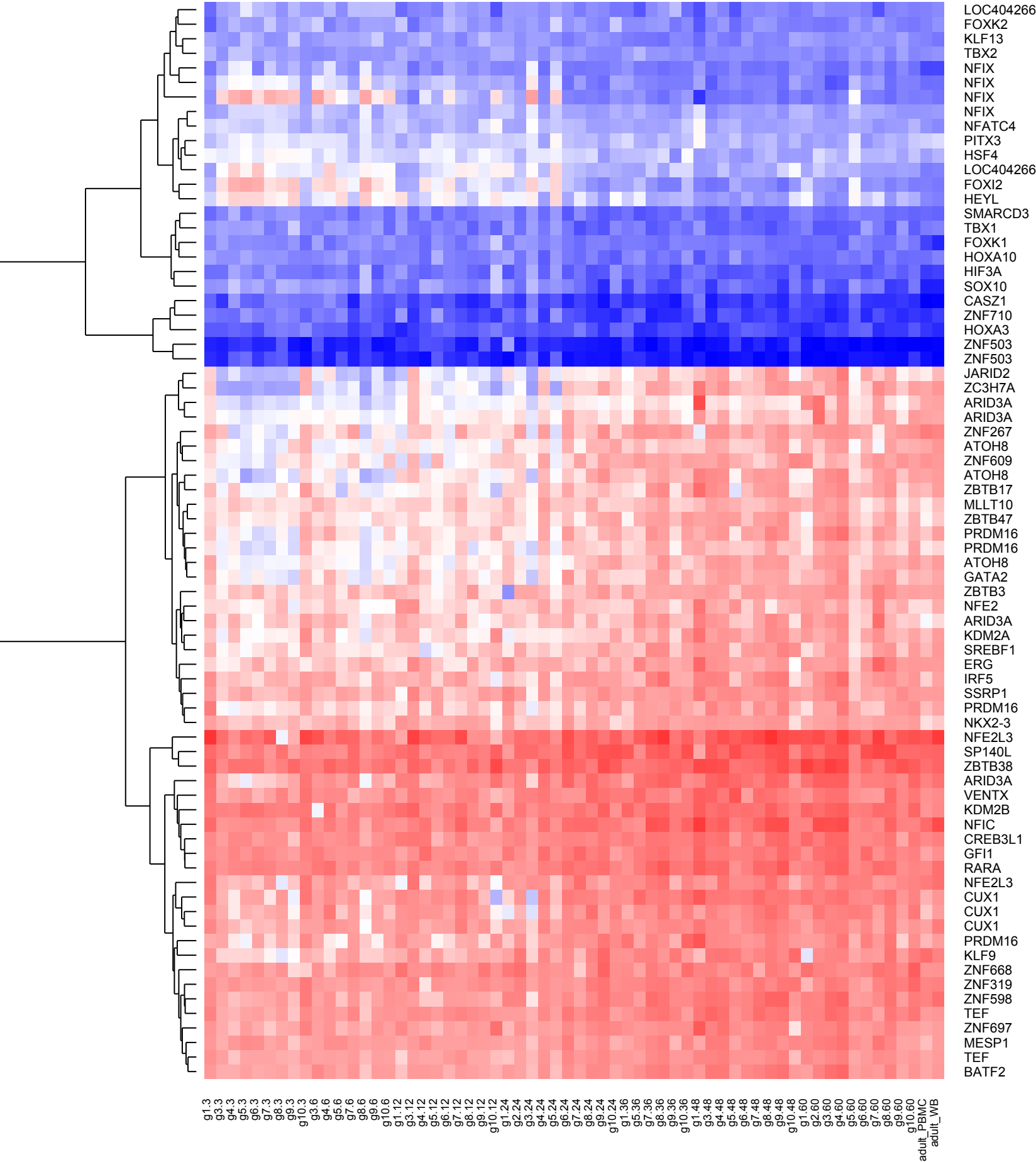

Supplement: Additional file 7: — DNA methylation levels in age-modified CpGs located on transcription factors and chromatin binding proteins. The histogram represents the M value: blue (methylated) and red (demethylated). A comparison with adult blood (WB) and sorted leukocytes as described by [34] is included for comparative purposes. PBMC = peripheral blood mononuclear cells. Each individual girl is represented by a column from 3 months (left) to 60 months (right). [file 13148_2015_64_MOESM7_ESM.pdf]
